# CFDP1 promotes hepatocellular carcinoma progression through activating NEDD4/PTEN/PI3K/AKT signaling pathway

**DOI:** 10.1002/cam4.4919

**Published:** 2022-07-21

**Authors:** Yan Zhou, Jiannan Qiu, Siyuan Liu, Peng Wang, Ding Ma, Guang Zhang, Yin Cao, Lili Hu, Zhongxia Wang, Junhua Wu, Chunping Jiang

**Affiliations:** ^1^ Department of Hepatobiliary Surgery Drum Tower Clinical College of Nanjing Medical University Nanjing China; ^2^ Department of Hepatobiliary Surgery The Affiliated Drum Tower Hospital of Nanjing University Medical School Nanjing China; ^3^ Jiangsu Key Laboratory of Molecular Medicine National Institute of Healthcare Data Science at Nanjing University, Medical School of Nanjing University Nanjing China; ^4^ Jinan Microecological Biomedicine Shandong Laboratory Shounuo City Light West Block Jinan City China

**Keywords:** CFDP1, hepatocellular carcinoma, NEDD4, PI3K/AKT signaling pathway

## Abstract

**Background and Aims:**

It is being increasingly reported that the Cranio Facial Development Protein 1 (CFDP1) plays a significant role in the onset and progression of tumors. Nonetheless, the underlying mechanisms associated with CFDP1 that contribute to hepatocellular carcinoma (HCC) and the specific biological role of CFDP1 remain vague.

**Methods:**

The Gene Expression Omnibus (GEO) database was analyzed to obtain the gene expression profiles as well as the matching clinical data of HCC patients. The gene co‐expression network was developed by means of weighted gene co‐expression network analysis (WGCNA) to screen for possible biomarkers that could be used for the purpose of predicting prognosis. The Cancer Genome Atlas (TCGA) and Gene Expression Profile Interaction Analysis (GEPIA) databases were used to assess the relationship between survival and expression. In addition, we identified the underlying mechanism associated with CFDP1 by analyzing the KEGG pathway database, applying the GSEA and GeneCards analysis method. We performed a sequence of experiments (in vivo and in vitro) for the purpose of investigating the specific function of CFDP1 in liver cancer.

**Results:**

The obtained results revealed high expression of CFDP1 in HCC tissues and cell lines. A positive correlation between the overexpression of CFDP1 and the adverse clinicopathological features was observed. Moreover, we observed that the low recurrence‐free survival and overall survival were associated with CFDP1 overexpression. In addition, GeneCards and GSEA analysis showed that CFDP1 may interact with NEDD4 and participate in PTEN regulation. Meanwhile, CFDP1 can promote the malignant development of liver cancer in vivo and in vitro. The western blotting technique was also employed so as to examine the samples, and the findings demonstrated that CFDP1 enhanced the malignancy of HCC via the NEDD4‐mediated PTEN/PI3K/AKT pathway.

**Conclusion:**

We highlighted that CFDP1 played an oncogenic role in HCC and was identified as a possible clinical prognostic factor and a potential novel therapeutic target for HCC.

## Graphical abstract

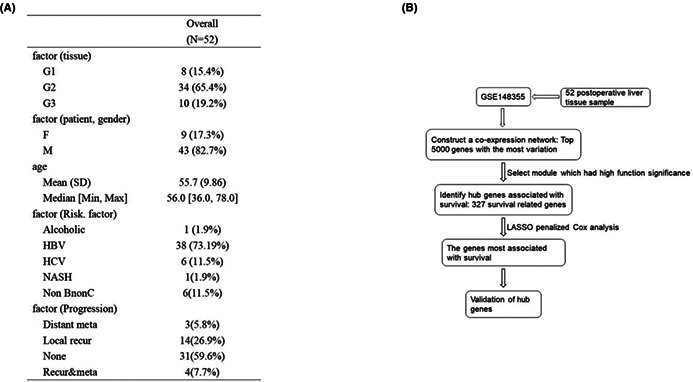

## 1 INTRODUCTION

Hepatocellular carcinoma (HCC) has been identified to be among the most lethal tumors, and the increasing extent in terms of the incidence rate of HCC is higher than that of all other tumors.^1^ The data acquired in 2018 by the Global Cancer Surveillance Agency reveals that HCC is one of the top five cancers on a global scale. The mortality rate caused by HCC is the highest among them, accounting for 8.2 percent of cancer‐related deaths.^2^, ^3^ It is estimated that in 2018, approximately 841,000 new cases of HCC were reported worldwide, and approximately 782,000 HCC patients died.^4^ The advancement in surgical techniques has made effective partial hepatectomy and liver transplantation possible, which are considered the mainstream treatment methods at present.^5^ However, high recurrence and metastasis rates following surgery severely affect the survival of HCC patients.^6^ Therefore, it is crucial to comprehend the occurrence and progression mechanisms of HCC to increase the chances of survival of patients suffering from HCC.

Numerous transcriptome and disease‐specific biomarker genes have been identified for the purpose of exploring the processes of occurrence and progression of HCC^7^. The high‐throughput platform has emerged as a promising tool that can be used for genomic analysis in the field of oncology.^7^ The expression of genes is being increasingly analyzed with the aid of this high‐dimensional data.^7^, ^8^, ^9^ Based on the results obtained using these techniques and bioinformatic analysis technology and the results reported in the literature, we found that the gene most related to prognosis was CFDP1.

Cranio Facial Development Protein 1 (CFDP1; family: evolutionarily conserved Bucentaur [BCNT] protein; length: 299 amino acids) contains the highly conserved C‐terminal BCNT domain1,2.^10^ The biological function of CFDP1 is unclear. However, studies have shown that the protein is involved in the maintenance of higher‐order chromatin tissue and influences the progression of the cell cycle.^11^ Previous research reports have indicated that blocking the activity of CFDP1 with antibodies results in decreased cell viability, increased number of apoptotic cells, and reduced the extent of proliferation.^12^ In clinically relevant disease studies, CFDP1 was identified as a new candidate pancreatic cancer susceptibility gene. The overall survival rate was reduced remarkably in patients when high levels of expression were observed.^13^ CFDP1 was listed as a candidate gene whose expression was regulated by risk variants in Esophageal adenocarcinoma (EA) and Barrett's esophagus (BE) tissues.^6^

We report that CFDP1 is closely correlated with the prognosis of HCC and is upmodulated in HCC tissues and cell lines. The progression of HCC was enhanced by CFDP1. The progression of HCC mediated by CFPD1 involves the processes of migration, proliferation, epithelial‐mesenchymal transition (EMT), invasion, and metastases observed in the cell lines. The inhibition of apoptosis also contributed to this process. The results revealed that CFDP1 is a potential prognostic biomarker that can be applied as an innovative treatment target for HCC.

## 2 MATERIALS AND METHODS

### 2.1 Materials

The American Type Culture Collection (Manassas, VA, USA) supplied six human HCC cell lines (SMMC7721, Focus, Huh7, HepG2, LM3, and Hep3B) and the normal liver cell line L02.

### 2.2 Data processing

GSE148355 was downloaded from the GEO database (submitted by: Jin‐Wu Nam). We selected 5000 genes with the most variation for the purpose of creating a co‐expression network. The human malignancy samples whose survival time was less than 30 days were ignored. Subsequently, we extracted genes from the expression profile, averaged the expression values of genes with the same symbol, and deleted the genes whose expression level was less than 30%. We selected the intersection of the expression and mutation data for the purpose of obtaining the intersection sample information of the expression and mutation data. We employed the Limma package (R software) to obtain the differentially expressed genes, and the threshold was |logfc| ≥ |log1.3| & *p* < 0.05.

### 2.3 Establishment of co‐expression module

The availability of the 5000 genes selected was studied. Certain cases whose complete clinical data could not be obtained were excluded. The WGCNA package (an R software package) was utilized for the purpose of constructing the gene co‐expression network. The following is the formula for the adjacency matrix AMN:

$$A_{\mathrm{mn}}={\left|S_{\mathrm{mn}}\right|}^{\beta }$$

where Aij denotes the contiguity of gene n and gene m, and S_mn_ denotes the Pearson's correlation between gene n and gene m. The value of β was set as 6 (soft threshold parameter; scale‐free R2 = 0.89). The adjacency matrix was transformed into the topological overlap matrix (TOM). In order to classify genes exhibiting greater absolute correlation, the difference measure derived from TOM was used for the purpose of classifying them into gene modules.

### 2.4 Identification of modules

The correlation between the clinical characteristics and the module characteristic genes was determined so as to comprehend the relationship between the module and clinical characteristics of HCC. The module significance of each module was also determined. It is generally believed that the modules with higher absolute significance have greater biological significance.

### 2.5 Hub genes: Identification and validation

The least absolute shrinkage and selection operator (LASSO) regression algorithm was applied for the purpose of identifying the candidate genes. The process of penalty parameter tuning (performed by 10−fold cross−validation with “survival” and “glmnet” packages) was used for conducting sample analysis. The LASSO Cox regression method was employed to determine the variables for constructing the signature and assigning the coefficients. The risk score, a measure of the prognosis of HCC patients, was computed as follows:

Risk score = expression level of Gene 1 × β1 + expression level of Gene 2 × β2 + expression level of Gene 3 × β3 + expression level of Gene 4 × β4, where β is the regression coefficient of each variable.

Subsequently, we verified their effect on survival (TCGA database) and picked out the genes that had an impact on survival. The ONCOMINE microarray database (https://www.oncomine.org) was utilized to examine the expression level of mRNAs of the screened hub genes. Comparisons were performed between HCC and non‐tumor liver tissues. Notably, the gene rank denotes the median ranking of a single target gene. The rank is determined by conducting several studies and analyzing all results (threshold: *p* = 0.05; 2‐fold change; top 10% gene rank). The UCSC Xena browser was used for the purpose of obtaining the hierarchical clusters of the hub genes present in primary HCC (TCGA HCC, n = 438). Data corresponding to 9736 tumors and 8587 controls are incorporated into the GEPIA database. The expressions of mRNAs of each hub gene present in liver hepatocellular carcinoma (LIHC) and non‐cancerous liver samples were determined by analyzing this comprehensive database. The website Human Protein Atlas (HPA) was utilized to delve into the expression levels of the proteins.

### 2.6 Gene enrichment, GSEA, and GeneCards analysis

The website http://www.geneontology.org, a Gene Ontology (GO) project, was accessed so as to acquire information on gene product function. This resource was exploited for the purpose of investigating ontologies and obtaining biological knowledge. We used the database to analyze gene GO and KEGG pathways. The GO project can be effectively used to obtain comprehensive knowledge of functional genomics. Evidence‐supported annotations can be obtained to define the biological functions of distinct genomic products (e.g., proteins, genes, various complexes, and ncRNAs). The results can be determined by means of classifying them using the ontologies obtained by us. The GO terms can be classified into three divisions: Cellular component (CC), molecular function (MF), and biological process (BP). Statistical significance was established as *p* < 0.05.

Gene Set Enrichment Analysis (GSEA) is a computational method that determines whether an a priori defined set of genes shows statistically significant, concordant differences between two biological states (e.g., phenotypes). And the GeneCards (https://www.genecards.org/) website can be accessed for the purpose of obtaining detailed information on annotated and predicted human genes. Relevant data from approximately 150 internet sources are collated to develop this comprehensive repository. Information on genomics, transcriptomes, proteomes, genetics, clinical methods, and functional information is integrated into this database. We used its online analysis capability to obtain its association with other differential genes.

### 2.7 Tissue samples and cell lines

Liver tissues (tumor and adjoining normal samples) were collected from 60 patients subjected to partial hepatectomy at the Department of Hepatobiliary Surgery of Nanjing Drum Tower Hospital. A part of the tissue sample was treated and stored in a solution of formaldehyde before conducting the experiment. Liquid nitrogen was used for the purpose of freezing the other portions of the tissues. This study was approved by the Ethics Committee of the Drum Tower Clinical College of Nanjing Medical University (2016‐057‐01) and conducted in accordance with the Helsinki Declaration and government policy. All participants signed a written informed consent form. All cells were treated with the Dulbecco's Modified Eagle Medium (DMEM; Gibco) which contained 10 percent fetal bovine serum (FBS) and 1% antibiotics (streptomycin/penicillin; Gibco). The samples were subjected to treatment at 37 °C. A humidified incubator (5% CO_2_) was used for sample treatment.

### 2.8 Lentiviral transfection

We purchased a Lentiviral vector encoding CFDP1 (LV‐CFDP1), short hairpin RNA (LV‐shCFDP1) against CFDP1, and the matching empty vector (LV‐control/LV‐shNC) from GenePharma Biotech. Polyamine (Genepharma; 5 μg/ml) was added into the cell lines with the aim of improving the efficiency of infection. The stably transfected cell lines were selected and added with 6 μg/ml puromycin for seven consecutive days.

### 2.9 Western blotting

The RIPA buffer containing 1% protease inhibitor cocktail (Cwbio, China) and 1% PMSF was used for the purpose of extracting the total protein of the tissues and cell lines. The samples were incubated on the ice (time: 30 min). Subsequently, the sample was subjected to centrifugation over a period of 15 min at a rate of 14,000× g. The sodium dodecyl sulfate‐polyacrylamide gel electrophoresis technique was utilized to isolate the proteins. Then, they have loaded onto polyvinylidene difluoride (PVDF) membranes (Millipore, USA). Blocking was performed using quick confining liquid (Beyotime). The blocking time was 30 min. Subsequently, incubation of the PVDF membranes was accomplished utilizing specific primary antibodies throughout the night at 4 °C. The horseradish peroxidase‐coupled secondary antibody (1:2000, Abcam) was used for incubation (2 h; ambient temperature). Furthermore, an enhanced chemiluminescence (ECL) reagent (Engreen, Beijing, China) was employed for the purpose of detecting the samples. Detection of the optical density of the protein bands was achieved with the aid of the ECL Western blot detection kit (Bio‐Rad). The antibodies used were listed as follows: CFDP1 (Abcam; Proteintech), NEDD4 (Proteintech), PTEN, AKT, p‐AKT, E‐cadherin, Vimentin, Bax, Bcl‐xl, Bcl‐2, GAPDH and horseradish peroxidase (HRP)‐conjugated anti‐rabbit IgG antibodies (Cell Signaling Technology).

### 2.10 Cell Counting Kit‐8 assay

Cell proliferation potential was examined with the help of the Cell Counting Kit (CCK‐8) (Vazyme Biotech Co., Ltd). The guidelines stipulated by the manufacturer were followed to conduct the studies. The cells were transferred to 96‐well plates (500 cells/well), and CCK‐8 solution (10 μl/well) was introduced into the wells. The samples were subjected to incubation for 2 h, following which the absorbance of each well was determined (at 450 nm).

### 2.11 Clone formation assay

First of all, cells in each group in the logarithmic growth phase were extracted and used for the preparation of cell suspensions. Subsequently, the process of gradient dilution was followed to prepare the samples, which were then inoculated into dishes containing culture medium (time: 2 weeks). The process was stopped when visual clones started appearing in the dishes. The supernatant was discarded, and the sample was subjected to fixing with 4% paraformaldehyde. Staining was performed using a solution of crystal violet (staining time: 30 min). Finally, the rate of clone production was computed, and the results were statistically examined.

### 2.12 Apoptosis assay

The apoptotic cells were identified with the aid of the Annexin V‐FITC/ Propidium iodide (PI) apoptosis detection kit (Vazyme Biotech Co., Ltd). The instructions provided by the manufacturer were followed to conduct the studies. Trypsin was used for the purpose of isolating the stable cell lines. EDTA was not used during the process. The sample was centrifuged at a rate of 1000 rpm at 4°C for 5 min, pre‐cooled with PBS, and washed twice. Subsequently, the sample was repeatedly centrifuged to form pellets. Cell precipitates were collected before suspension using 100 μl of a combined buffer. Annexin V labeled FITC (5 μl) and PI (5 μl) were introduced into the cell suspension, and the reaction was allowed to proceed (15 minutes; ambient temperature; darkness). Ultimately, the binding buffer (400 μl) was introduced into the cell suspension, and the solutions were mixed gently. The flow cytometry technique was used for the purpose of determining the apoptosis level (time: 1 h). The data on apoptosis was examined with the aid of the FlowJo software.

### 2.13 Transwell assay

The migratory and invasive abilities of the cells were studied utilizing the Transwell chamber (Millipore, USA). The cell lines were implanted into the upper lumen. The samples were subjected to culturing using 200 μl of DMEM devoid of FBS. The lower lumen was filled with 500 μl of the complete medium which contained 10% fetal bovine serum. The cells were induced through the Transwell membrane into the bottom lumen. The cells were incubated for 1 day, following which we discarded the medium. The Transwell Chambers were rinsed two times using PBS. We then fixed the cells using methanol over a period of 20 min. Crystal violet (0.1%) was used for staining the invasive and migratory cells (room temperature; dark; 30 min). The stained cells were rinsed thrice using PBS and observed with the aid of a light microscope.

### 2.14 Wound‐healing assay

HepG2 and Hep3B and cell lines (density: 5 × 10^5^ cells/well) were implanted into 6‐well plates. Once the cells reached the conditions of fusion, they were rinsed twice with PBS. Uniform linear scratches were made at the middle of the hole using a sterilized pipette tip (volume: 200 μl). The distance between the wound edges was recorded 0 h following two cycles of PBS washing. The result was taken as the baseline. The images of the same location were obtained 48 h later. Changes in the distance were determined with the aim of investigating the extent of cell migration and repair realized.

### 2.15 Extraction of RNA and qRT‐PCR assay

The Easypurure RNA kit (Regent bio) was utilized to isolate the total RNA. The guidelines stipulated by the manufacturer were followed to conduct the experiments. In short, a lysis buffer was used for the purpose of mixing the tissue or cells. Following effective lysis, the lysis fluid was collected and swirled for 10 s. Subsequently, an equal amount of anhydrous ethanol was added to the mixture. The lysate was transferred to an RNA column. A centrifuge tube was used, and the process of centrifugation was conducted at a rate of 4000 *g* and a temperature of 4°C. A clean buffer (500 μl) was used, and the centrifugation time was 1 min. Further, the sample was centrifuged at 12000 *g* for 1 min. RNAse‐free tubes were used in the latter part of the experiments. The RNA columns were transferred to new and RNAse‐free tubes, and the tubes were subsequently air‐dried over a period of 2 min. The central column of RNA was subjected to treatment with the elution buffer (20 μl), and the waiting time was 2 min (centrifugation conditions: 12000 g; 1 minute). The extracted RNA was preserved at a temperature of −80°C for the purpose of conducting further experiments. The reaction mixture (20 μl/well) consisted of total RNA (1 μg), 5 × HiScript IIqRT SuperMix (4 μl; Vazyme Biotech Co., LTD), and ddH2O.

We used the ChamQTM Universal SYBR qPCR Master Mix (Vazyme Biotech Co., Ltd) system to perform studies using the qRT‐PCR technique. Primer sequences used were as follows: CFDP1: Forward Sequence 5′‐CCACAGGCTAATGTTCCTTCAGC‐3′ and Reverse Sequence 5′‐GTCCAGTTTGGACTTCTCAAGGG‐3′. β‐actin: 5′‐TGACGTGGACATCCGCAAAG‐3′ (forward) and 5′‐CTGGAAGGTGGACAGCGAGG‐3′ (reverse).

### 2.16 Immunohistochemical (IHC) staining

A solution of 4% formaldehyde was used to fix the samples, followed by embedding in paraffin. The paraffin system was cut into 4‐μm slices, and CFDP1 and ki‐67 (Abcam) primary antibody was used for conducting sample incubation at 4°C (incubation time: 12 h). Following this, the slices were subjected to incubation at ambient temperature with the aid of HRP‐conjugated secondary antibody over a period of 1 h. The samples were detected following 3,3′‐diaminobenzidine and hematoxylin staining methods. Staining positivity and intensity were assessed in a blind study by two independent pathologists. Ten areas were randomly selected from each slide for the purpose of conducting the experiments.

### 2.17 5‐Ethynyl‐2′‐deoxyuridine (EdU) assay

The EdU test kit (RiboBio, Guangzhou, China) was employed for the purpose of determining the ability of the cell lines to proliferate. The guidelines stipulated by the manufacturer were followed to conduct the experiments. 96‐well plates were used for planting the cell lines (density: 2 × 104 cells/well). The complete medium was used to culture the samples (time: 24 h). EdU (50 μmol/L; RiboBio) was used to incubate the cells (incubation time: 2 h; incubation temperature: 37 °C). with a solution of 4% formaldehyde was used for the purpose of fixing the cell lines (time: 30 min). TritonX‐100 (0.5%) was used to permeabilize the cell lines (time: 10 min). The cell lines were rinsed thrice with PBS. Subsequently, the 1 × ApolloR reaction cocktail (400 μl) and Hoechest33342 (400 μl) were used for the visualization of the EdU‐positive cells. Finally, the images of the cells were recorded under a microscope.

### 2.18 TUNEL assay

The TUNEL staining technique was utilized for the detection of the DNA fragmentation characteristics of apoptosis in formalin‐fixed tumor slices embedded in paraffin. The Klenow‐Fragel DNA fragment detection kit (Roche, Basel, Switzerland) was used, and the protocols outlined by the manufacturer were followed. Ten areas were randomly selected from each slide for examination.

### 2.19 Animal‐based experiments

Approval of all animal‐based experiments was granted by the Institutional Committee of Animal Care and Use of Nanjing Medical University. Female BALB/c nude mice at an age of 4 weeks were procured from the Nanjing Medical University's Experimental Animal Center and were used in the present research. PBS (100 μl) was used to resuspend the transfected cell lines (1 × 106 cells). The suspension was subcutaneously injected into nude mice (*n* = 6 per group) to monitor the subcutaneous growth of tumors. For the purpose of detecting metastasis, a total of 1 × 106 luciferase‐expressing HCC cells were injected into the tail vein of nude mice (*n* = 12 per group). The IVIS100 imaging system (Caliper Life Sciences) was utilized to observe distant metastases after 5 weeks.

### 2.20 Statistical analysis

SurvMiner software package in R was used for the purpose of classifying the patients into low‐ and high‐risk groups. The method was on the basis of the maximum selective rank statistic. The optimum threshold expression value corresponding to each hub gene was considered. We analyzed relapse‐free survival (RFS) and overall survival (OS) utilizing the Kaplan–Meier method and compared the survival curves via the use of the Log‐rank test. GraphPad Prism (GraphPad Software, version 5.0) and R (version 4.0.3) were used to conduct the statistical analysis. *p* < 0.05 was established as the criterion for statistical significance.

## 3 RESULTS

### 3.1 Constructing the co‐expression module

**FIGURE 1:**
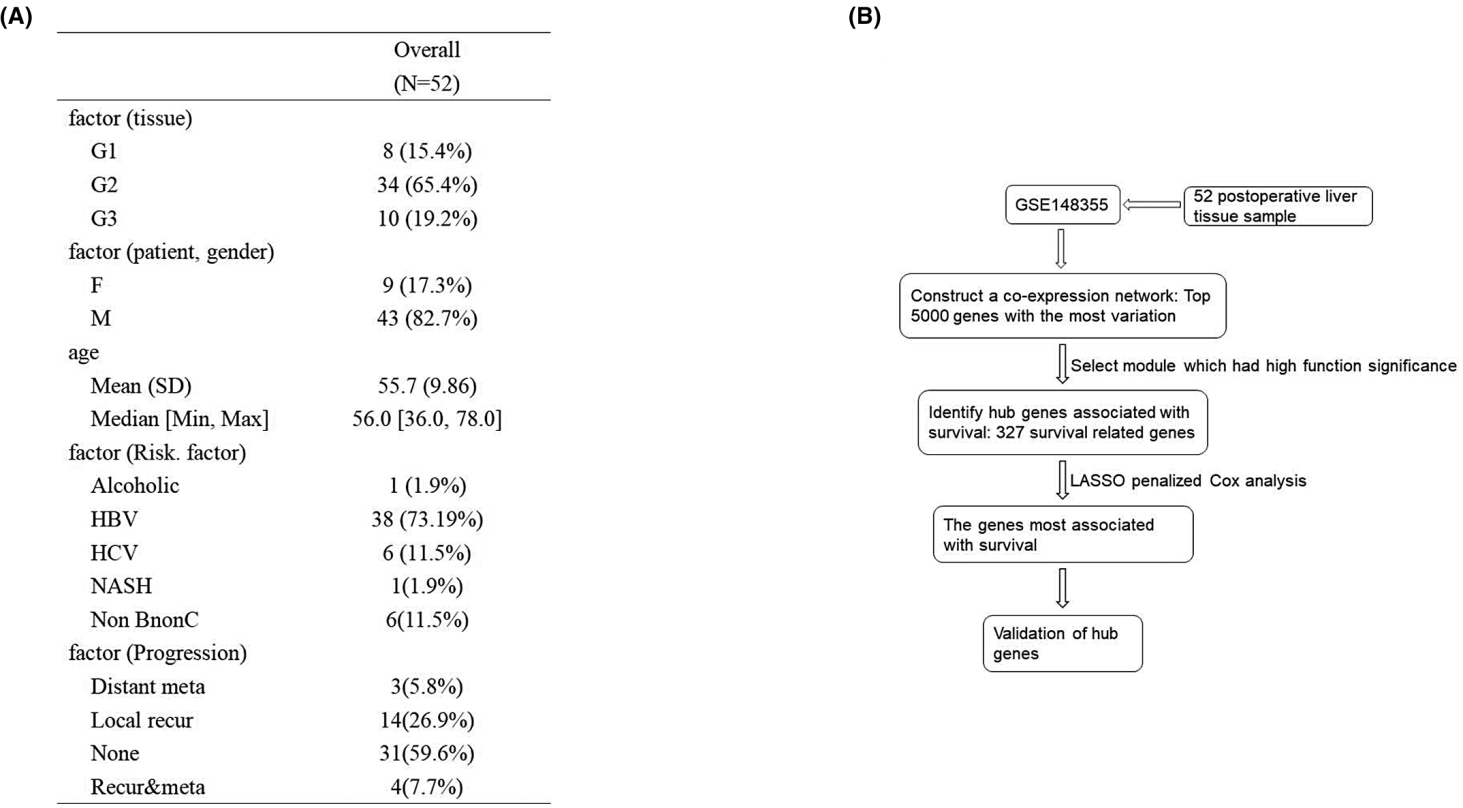
Data set sample description and Flow chart. (A) Clinicopathological features of patients with HCC. (B) Flow chart showing the target gene selection process followed by us

**FIGURE 2:**
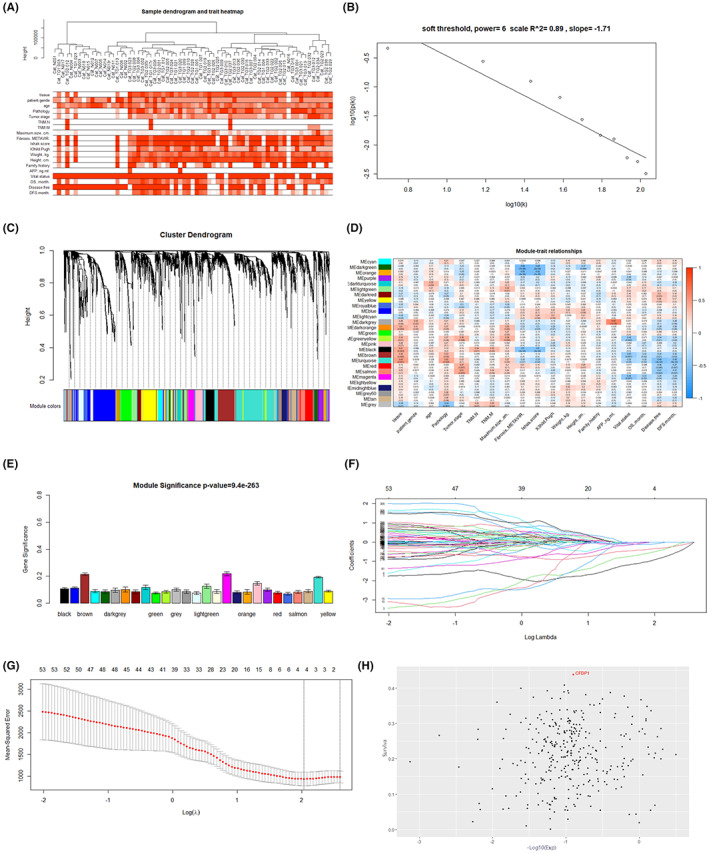
A dendrogram for clustering and the assessment of soft‐thresholding power (WGCNA); Determination of modules correlated with the clinical characteristics of HCC; Ten‐time cross‐validation for the selection of tuning parameter and survival correlation. (A) Clustering dendrogram generated for 52 samples. (B) Mean connectivity analysis for different soft‐threshold powers. (C) Clustering of differentially expressed genes with the aid of dendrograms created using a dissimilarity measure(1‐TOM). (D) Relationship between module eigengenes and clinical characteristics visualized as a heatmap. € Mean gene significance and error distributions in modules correlated with HCC tumor grades. (F) Ten‐times cross‐validation for the selection of tuning parameters (lasso model). (G) LASSO coefficient profiles were recorded for the 327 prognostic genes. (H) Relationship between survival and expression of differential genes

The expression matrix corresponding to 52 samples was obtained from the data set GSE148355 (Figure 1A,B). We selected the first 5000 mutant genes to conduct co‐expression analyses. Cases, where complete clinical data were unavailable were excluded from the present research. Clustering of GSE148355 samples was performed utilizing the average linkage approach to determine the microarray's quality. The outlier differentially expressed gene samples were also filtered out using this method (Figure 2A,B). A power of β = 6 was set as the soft threshold (scale‐free R2 = 0.89). A total of 27 modules were constructed by means of the average linkage hierarchical clustering approach. Further analysis was conducted using the Brown module. Finally, we observed that the Brown module was remarkably correlated with the OS time (Figure 2C–E).

### 3.2 Hub gene identification

Three hundred and twenty seven genes were considered to be the key genes of the Brown module, which exhibited high functional significance. The LASSO regression analysis method was utilized to further narrow the range of genes that were used to construct the prognostic model. Subsequently, the differential gene most related to survival, namely CFDP1, was screened out in the Brown Module (Figure 2F–H).

### 3.3 CFDP1 expression is up‐regulated in HCC: Poor prognosis

**FIGURE 3:**
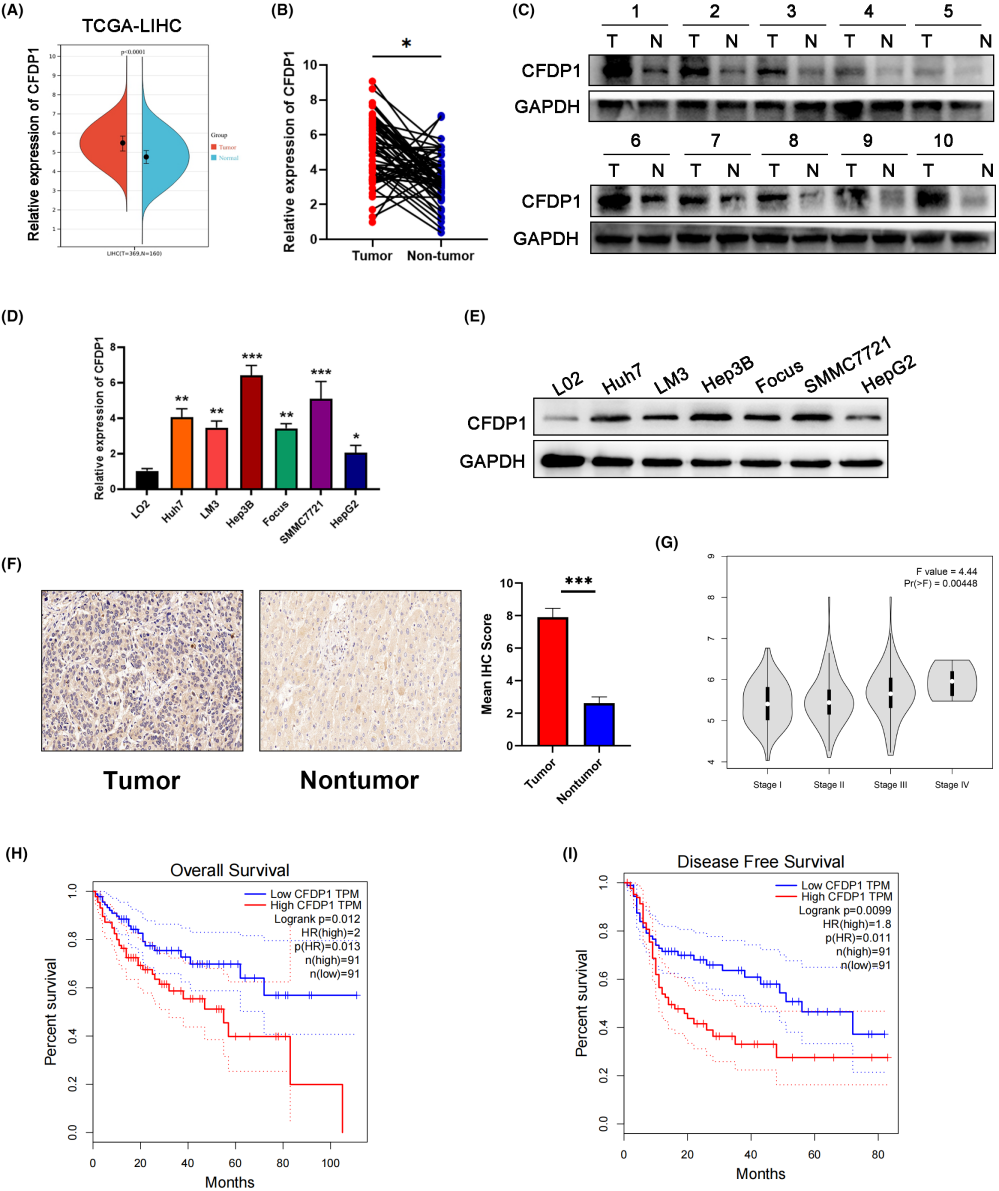
CFDP1 is overexpressed in HCC. (A) CFDP1 expression analysis using TCGA data in HCC and adjacent normal samples. (B) Quantitative RT‐PCR analysis was conducted for determining the expression of CFDP1 in HCC tissues and the corresponding normal samples. (C) Levels of CFDP1 in 10 random HCC tissue pairs were evaluated by means of the western blot analysis technique. (D, E) Quantitative analysis of relative levels of protein and mRNAs, conducted using the RT‐PCR and western blotting analysis techniques (for CFDP1 in the L02 cells and six human HCC cell lines). (F) CFDP1 expression in tumor and adjoining normal samples were examined by IHC and IHC score was quantified. (G) The relationship between CFDP1 expression and the HCC stage was analyzed by means of analyzing the GEPIA database. (H, I) Overall survival and recurrence‐free survival of CFDP1 were analyzed utilizing the GEPIA database. *p < 0.05, **p < 0.01, and ***p < 0.001

Data corresponding to RNA sequencing were downloaded from TCGA (369 HCC and 160 nontumor tissues) to probe into the expression profiles of CFDP1 in HCC. Analysis of the results revealed that the level of expression of CFDP1 in HCC tissues was remarkably elevated as opposed to that recorded for normal tissues (Figure 3A). To determine whether CFDP1 is significantly overexpressed in HCC samples, we used the qRT‐PCR technique to detect the relative mRNA levels of CFDP1 in 60 HCC pairs and the corresponding adjoining non‐tumor samples. The findings illustrated that the relative level of CFDP1 mRNA in HCC tissues increased significantly (Figure 3B). Additionally, 10 randomly selected HCC tissue pairs were analyzed by means of the western blot technique, and the results revealed the upregulation of CFDP1 (Figure 3C). The CFDP1 mRNA and protein relative levels increased in Huh7, HepG2, Hep3B, LM3, SMMC7721, and Focus HCC cell lines. The levels in these cases were higher than the levels recorded for the normal liver cell line LO2 (Figure 3D,E). The IHC results also revealed that the level of expression of CFDP1 in HCC samples was considerably elevated in contrast with the level of expression of CFDP1 recorded for the adjacent normal tissues, and the corresponding IHC Score also confirmed this (Figure 3F). Then, the relation between CFDP1 and survival and tumor stage was analyzed using the GEPIA database, an online bioinformatics tool. Moreover, we observed that the CFDP1 expression was correlated positively with the HCC stage. Patients suffering from HCC and characterized by high CFDP1 expression experienced a high recurrence rate and poor overall survival (Figure 3G–I). We classified 60 patients into high CFDP1 and low CFDP1 groups for the purpose of investigating the specific relationship between the expression of CFDP1 and the clinical‐pathological features of HCC patients. Analysis of the findings revealed that large tumor size, increased extent of microvascular invasion, and high TNM and Edmondson stages could be attributed to high levels of CFDP1 (Table 1). Overall, these results indicate the overexpression of CFDP1 under in vitro and in vivo conditions in HCC. These findings indicate that CFDP1 can play a potential role in the occurrence and progression of HCC. Thus, it can potentially be a prognostic biomarker of HCC.

**TABLE 1:** Association between CFDP1 expression and clinicopathological features in patients with HCC (*n* = 60)

Characteristics	Number		CFDP1 expression	*p*‐value
	Low group	High group		
Age(years)				
<50	33	17	16	0.7952
≥50	27	13	14	
Gender				
Female	23	10	13	0.4257
Male	37	20	17	
Cirrhosis				
Present	34	18	16	0.6023
Absent	26	12	14	
HBV infection				
Positive	43	21	22	0.7745
Negative	17	9	8	
Tumor size(cm)				
<5	35	22	13	0.0184^*^
≥5	25	8	17	
Microvascular invasion				
Presence	38	12	26	*p* < 0.001^***^
Absence	22	18	4	
Tumor multiplicity				
Simple	22	14	8	0.108
Multiple	38	16	22	
α‐fetoprotein (ng/ml)				
≤20	20	11	9	0.5839
>20	40	19	21	
TNM stage				
I	37	25	12	*p* < 0.001^***^
II/III	23	5	18	
Edmonson stage				
I/II	39	26	13	*p* < 0.001^***^
III/IV	21	4	17	

^*^ *p* < 0.05,

^***^ *p* < 0.001.

### 3.4 CFDP1 promotes the progression of HCC malignancy

**FIGURE 4:**
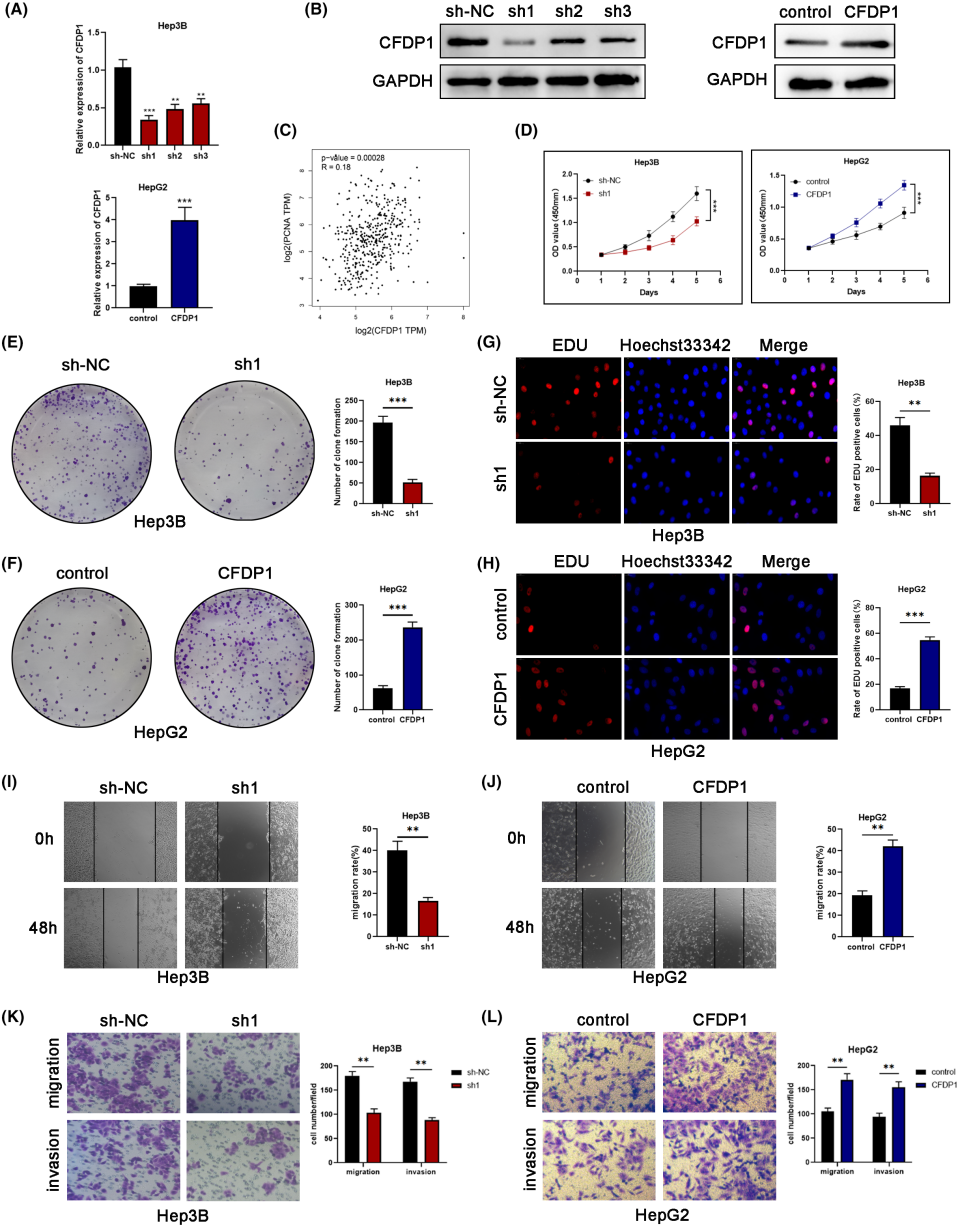
CFDP1 influences the ability of HCC cells to proliferate, migrate, and invade. (A, B) The effectiveness of CFDP1 silencing and overexpression as determined with the aid of the qRT‐PCR and western blotting technique (C) Relationship between BUB1B and PCNA examined utilizing the GEPIA database. (D–H) Colony formation, CCK‐8, and EDU assays for the assessment of the cell proliferation in HCC cell lines that were knocked down or overexpressed for CFDP1. (I, J) Wound‐healing assays conducted for the purpose of detecting cell migration in cell lines characterized by the overexpression of knockdown of CFDP1 (K, L) Transwell assays conducted to determine the capability of HCC cell lines to migrate and invade under conditions of CFDP1 knockdown or overexpression. *p < 0.05, **p < 0.01, and ***p < 0.001

The results obtained using the western blot and qRT‐PCR techniques were subjected to analysis. Based on the results, the Hep3B cells were selected to conduct the CFDP1 gene knockdown experiment. This is because Hep3B cells expressed a high level of CFDP1 among the six detected HCC cell lines. Meanwhile, HepG2 cells characterized by low levels of CFDP1 expression were selected to conduct the CFDP1 overexpression experiment. The highest knockout efficiency was taken into consideration, and the results obtained using the qRT‐PCR and western blotting technique were analyzed. According to these findings, sh1 was selected to conduct further experiments. Upregulation of the expression of CFDP1 was observed in the HepG2 cells transfected with Lv‐CFDP1. Hence, it confirmed the overexpression efficiency of CFDP1 (Figure 4A,B).

Firstly, we evaluated the correlation between CFDP1 and the proliferating cell nuclear antigen (PCNA). We discovered a positive correlation between the CFDP1 expression and PCNA (Figure 4C). The results obtained by conducting the CCK‐8 assay indicated that the down‐regulation of CFDP1 expression could reduce the proliferation ability of the Hep3B cells (Figure 4D). Furthermore, following the knockdown of CFDP1, the clonal survival of Hep3B cells was reduced significantly (Figure 4E). The results were in line well with the previously obtained results. Considering the specificity and sensitivity of CFDP1, we used the EdU incorporation method to evaluate its effect on the process of actual cell proliferation from multiple perspectives. The results revealed that the inhibition of CFDP1 could result in the inhibition of the proliferation of the Hep3B cells (Figure 4G). Following this, we performed wound healing and Transwell experiments to investigate the influence of CFDP1 knockdown on the migratory and invasive processes of HCC cells. As is shown in Figure 2I, the extent of migration of Hep3B cells reduced following CFDP1 knockdown. Results from Transwell experiments revealed that CFDP1 knockdown resulted in the inhibition of migration and invasion of the Hep3B cells (Figure 2K). To thoroughly comprehend the function of CFDP1, we used HepG2 cells with high expression of CFDP1 for EdU assay, wound‐healing assay, and Transwell experiments. As expected, overexpression of CFDP1 significantly enhanced the HepG2 cells' capability of proliferating, migrating, and invading (Figure 2H,J,L).

### 3.5 CFDP1 overexpression inhibits apoptosis and promotes EMT

**FIGURE 5:**
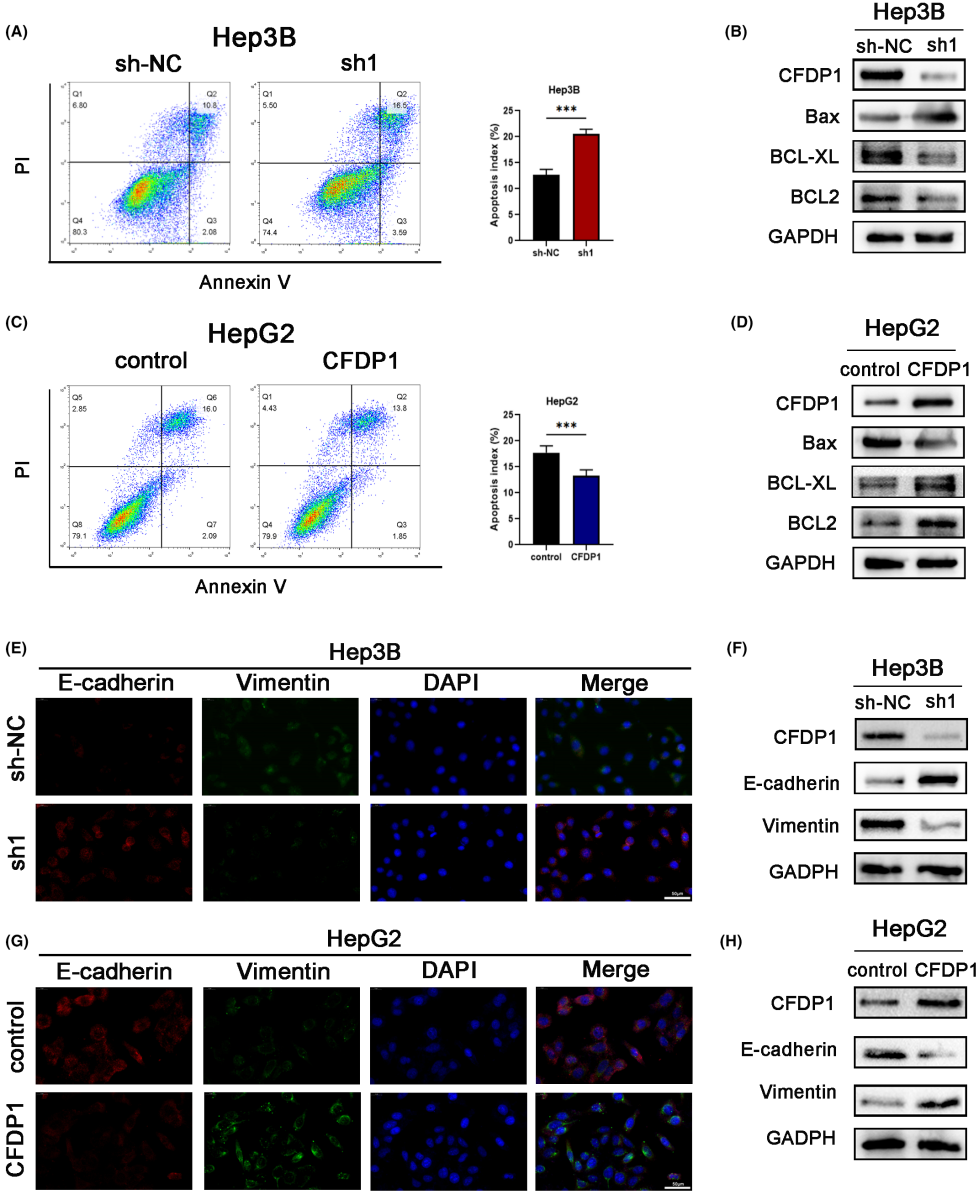
CFDP1 overexpression inhibits apoptosis and promotes the migratory and invasive capacity of cells via EMT. (A–D) Apoptosis of HCC cell lines under conditions of CFDP1 overexpression and silencing examined applying the Western blotting and flow cytometry methods for apoptosis‐related proteins in HCC cell lines characterized by CFDP1 knockdown or overexpression. (E–H) Immunofluorescence and western blotting utilized with the aim of examining the expression of CFDP1, Vimentin, and E‐cadherin in HCC cell lines with stably downregulated or overexpressed CFDP1. *p < 0.05, **p < 0.01, and ***p < 0.001

The rate of apoptosis plays a significant role in the occurrence and progression of HCC. The flow cytometry technique was employed to investigate the rate of apoptosis of cells. Our results suggest that the down‐regulation of CFDP1 promotes the apoptosis of the Hep3B cells, while the overexpression of CFDP1 reduces the extent of apoptosis of the HepG2 cells (Figure 5A,C). western blotting results showed that the expression level of apoptosis‐related protein Bax in Hep3B cells increased following the knockdown of CFDP1. It was also observed that the expression level of the antiapoptotic proteins BCL‐XL and Bcl‐2 decreased under these conditions. However, the opposite effect was observed in HepG2 cells characterized by overexpressed CFDP1 (Figure 5B,D).

We evaluated the expression of the “EMT master gene” and analyzed the results to check if CFDP1 enhances the ability of the cells to migrate and invade through EMT. The results obtained from immunofluorescence experiments revealed low levels of expression of Vimentin (mesenchymal marker) and high levels of expression of E‐cadherin (epithelial marker) in Hep3B‐sh1 cells. These results were in line with the results obtained from the western blotting (Figure 5E,F). Low levels of expression of E‐cadherin and high levels of expression of Vimentin (Figure 5G,H) were observed in the CFDP1 exhibiting overexpression of HepG2 cells. The above findings suggest that CFDP1 overexpression inhibits apoptosis and promotes EMT in HCC cell lines.

### 3.6 CFDP1 contributes to the growth of xenograft tumors and liver cancer metastasis in vivo

**FIGURE 6:**
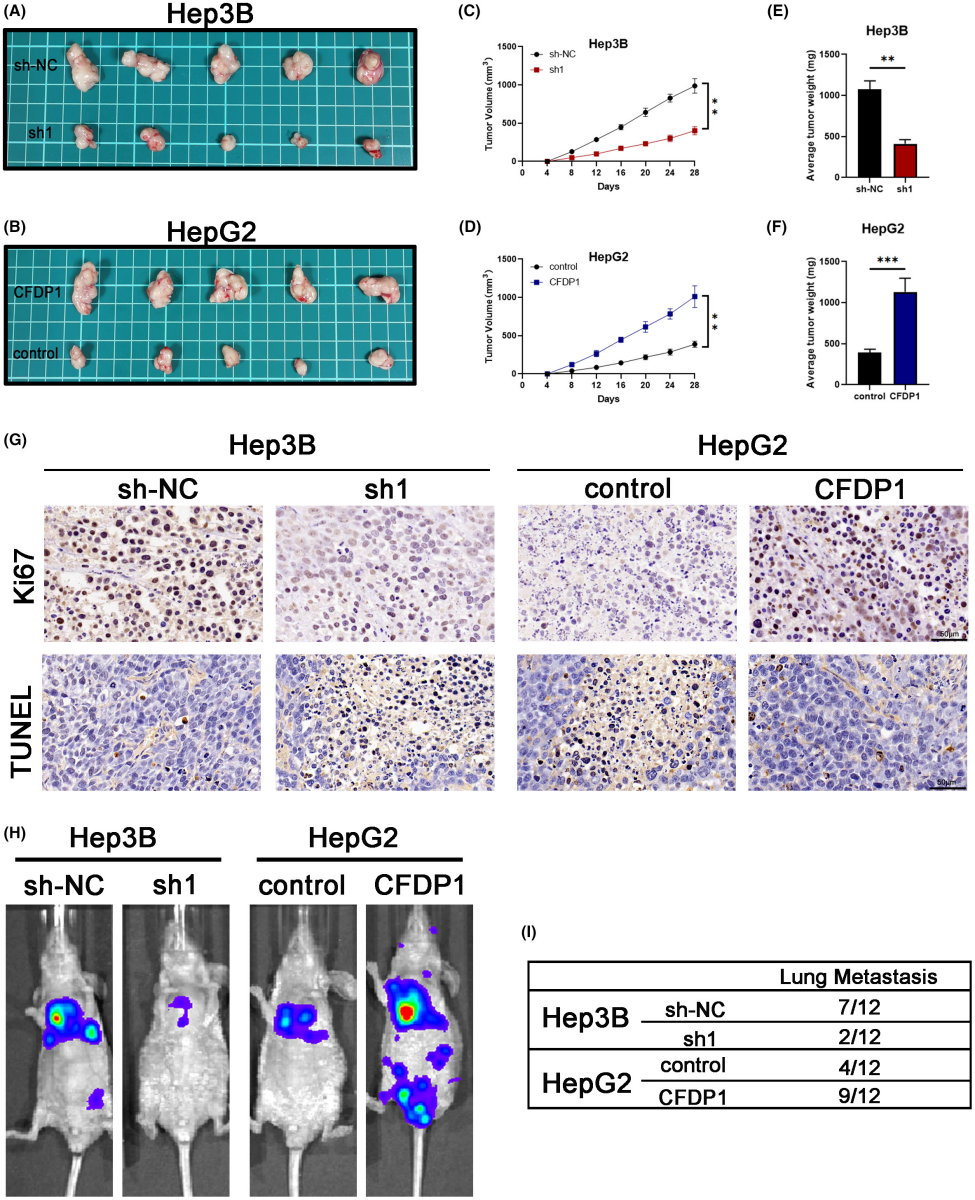
CFDP1 promotes the progression and metastasis of HCC in vivo. (A, B) Typical images of transplanted HCC tumors taken from mice undergoing treatment with an injection of Hep3B‐SH1 and Hep3B‐shNC or HepG2‐CFDP1 and HepG2 controls. (C, D) The tumor volume growth curves of each group were calculated. (E, F) Tumor weight of HCC in each group. (G) IHC images of KI‐67 in each group and TUNEL expression in xenograft HCC tumor. Scale: 50 μm. (H) bioluminescent imaging. (I) The table indicates the proportion of mice that developed lung metastases. (I) Data are expressed as the mean ± SD of a minimum of 3 separate trials. *p < 0.05, **p < 0.01, and ***p < 0.001

For the purpose of thoroughly exploring the impact of CFDP1 on tumor growth, we generated subcutaneous xenograft tumor mouse models of HCC. The rate of growth of tumors was slow in mice injected with the Hep3B‐sh1 cells. The average tumor weight and volume were low in this case (Figure 6A,C,E). In contrast, the rapid growth of tumors was observed in mice undergoing treatment with an injection of HepG2 cells accompanied by overexpressed CFDP1. The average tumor size and weight were higher in this case as opposed to that recorded for the previous case (Figure 6B,D,F). These findings indicate that CFDP1 may increase tumor growth in vivo. The results obtained by performing TUNEL assays and Ki‐67 staining confirmed that CFDP1 enhanced tumorigenicity. The findings revealed that the hep3B‐sh1 xenograft exhibited lower proliferation activity and stronger apoptosis compared with Hep3B‐shNC. In contrast, up‐regulation of CFDP1 can result in increased proliferation activity and decreased apoptosis of HepG2 xenogeneic cells (Figure 6G). We injected the luciferase‐expressing and stably transfected liver cancer cells into BALB/C nude mice via tail vein to further investigate whether CFDP1 affects the metastasis of liver cancer (in vivo). The results were compared with the results obtained by studying the corresponding control group. According to the results, the downmodulation of CFDP1 in Hep3B cells played a role in the occurrence of few lung metastases. An increased extent of metastases was observed in the group overexpressing HepG2‐CFDP1 (Figure 6H). In each group, we counted the number of mice experiencing lung metastasis (Figure 6I).

### 3.7 CFDP1 interacted with NEDD4, promoting the growth and migration of HCC cells: The PTEN/PI3K/AKT pathway

**FIGURE 7:**
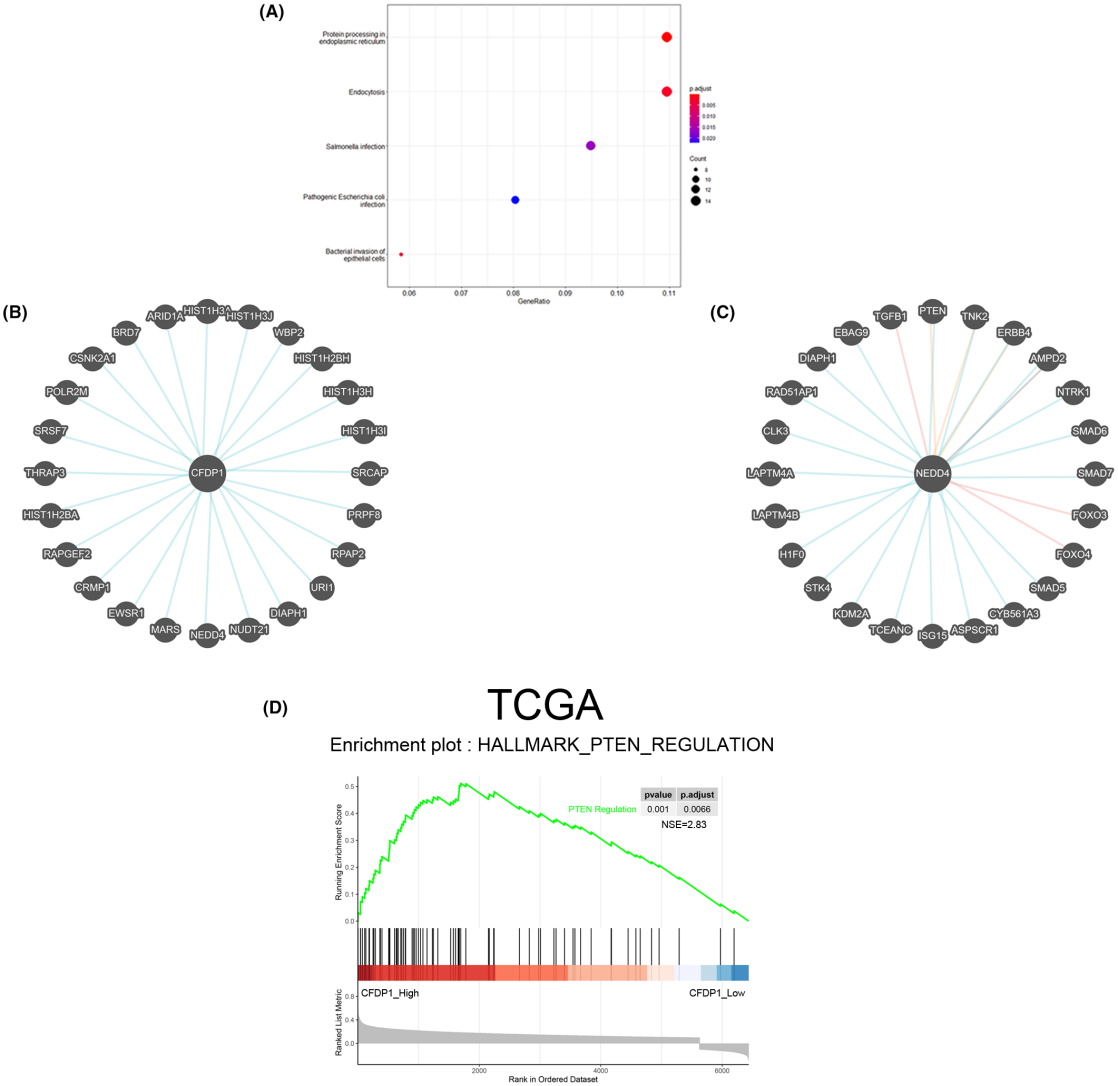
Pathway enrichment analysis of Brown module genes and GeneCards analysis. (A) KEGG pathway analysis. (B) Association of CFDP1 with potentially differential genes. (C) Association of NEDD4 with other differential genes. (D) GSEA analyzed the related signaling pathway influenced by CFDP1 based on TCGA data

**FIGURE 8:**
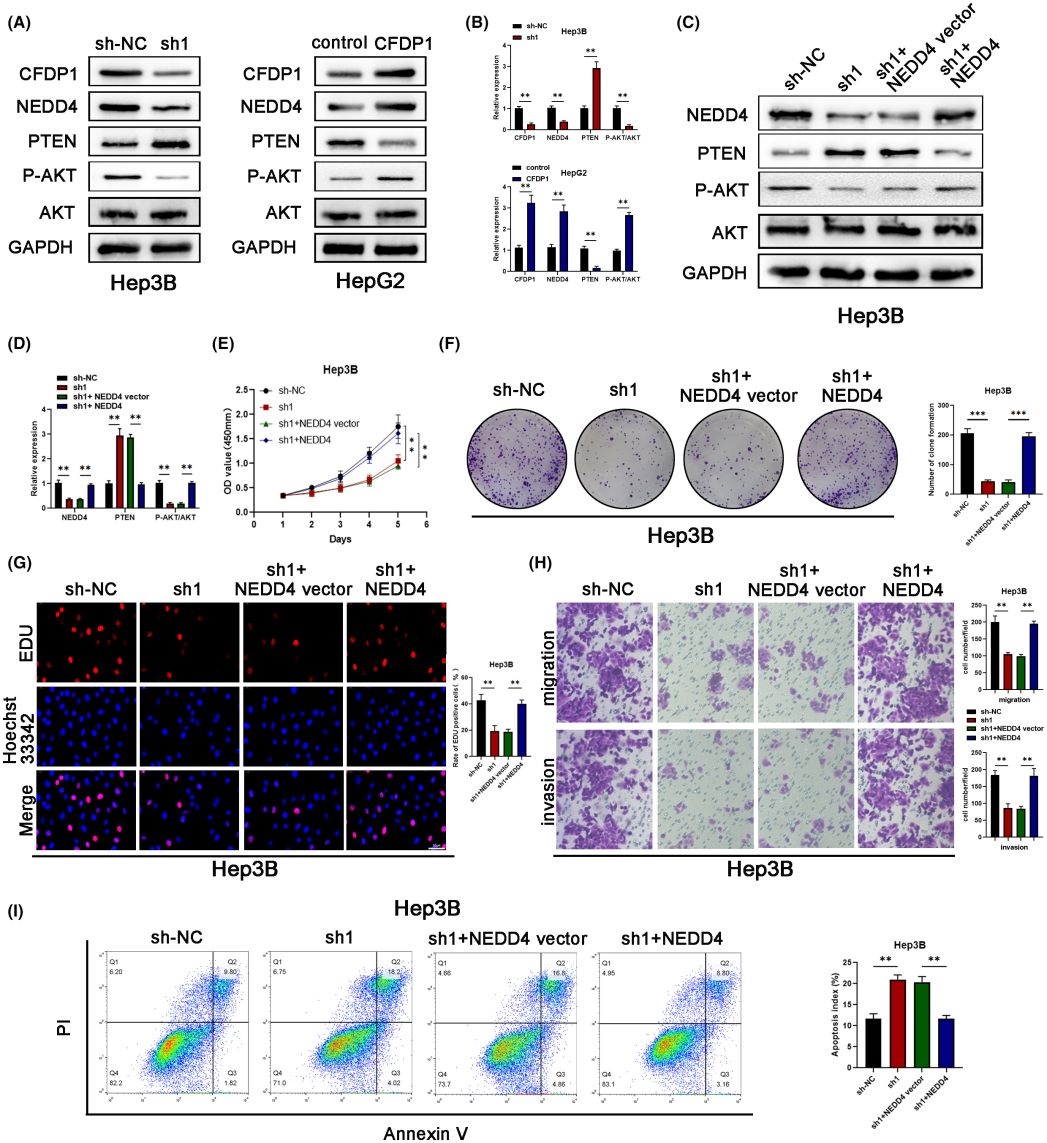
CFDP1 activates the expression of NEDD4 through the PTEN/PI3K/AKT pathway. (A, B) Western blot examination of numerous critical components, including CFDP1, NEDD4, and the PTEN/PI3K/AKT pathway. (C, D) Western blot analysis of the levels of a variety of critical components involving the NEDD4 pathway when Hep3B‐shNC and Hep3B‐shCFDP1 in the presence or absence of NEDD4 overexpression. (E‐I) CCK‐8, colony formation, EdU, Transwell, and flow cytometry were utilized for detecting the proliferation, invasion, migration, and apoptosis of Hep3B‐shNC and Hep3B‐shCFDP1 in the presence or absence of NEDD4 overexpression. *p < 0.05, **p < 0.01, and ***p < 0.001

We applied the Enrichment Analysis and GeneCards online tool to further determine the possible signaling pathway mediated by CFDP1. The results from enrichment analysis demonstrated that the genes in the Brown module were primarily enriched in protein folding and endocytosis (Figure 7A). Subsequently, as shown in Figure 7B, proteins that are potentially related to CFDP1 were obtained using the GeneCards online analysis tool. It has been reported that one of the main functions of NEDD4 is to regulate a large number of membrane proteins through its ubiquitination activity. It has the ability to mediate endocytosis and promote the growth as well as the migration of HCC cells via the PTEN/PI3K/AKT signaling pathway. In addition, GeneCards analysis also confirmed the relationship between NEDD4 and PTEN (Figure 7C). GSEA analysis results showed that CFDP1 was highly enriched in PTEN regulation (Figure 7D). Next, we explored the relationship between the CFDP1, NEDD4, and PTEN/PI3K/AKT signaling pathways. We detected several core proteins, including CFDP1, NEDD4, and the PTEN/PI3K/AKT pathway. In CFDP1 down‐regulated Hep3B cells, the protein expression of NEDD4 and P‐AKT was reduced, whereas the protein expression of PTEN was elevated. On the contrary, an opposite trend in the protein levels was observed in the CFDP1‐overexpressed HepG2 cells (Figure 8A,B). The obtained data suggest that CFDP1 can potentially promote the malignancy of HCC through the NEDD4/PTEN/PI3K/AKT pathway. Subsequently, the Hep3B‐sh1 cells were subjected to transfection with the LV‐NEDD4 (or LV‐NEDD4) vector to confirm whether the effect of CFDP1 depends on the NEDD4 pathway. Results obtained using the western blot technique revealed that the expression of PTEN was attenuated, and the expression of P‐AKT was elevated in the CFDP1‐downregulated Hep3B cells when the cells were transfected with LV‐NEDD4 (Figure 8C,D). The CCK‐8 results showed that NEDD4 overexpression increased the proliferation capacity of the CFDP1‐downregulated Hep3B cells (Figure 8E). In line with this, overexpression of NEDD4 significantly increased the clonal survival rate of the CFDP1‐downregulated Hep3B cells (Figure 8F). As shown in Figure 8G, results obtained from EdU experiments revealed that overexpressed NEDD4 promoted the proliferation of the CFDP1‐downregulated Hep3B cells. Results obtained from Transwell experiments illustrated that NEDD4 overexpression enhanced the migratory and invasiveness of the CFDP1‐downregulated Hep3B cells (Figure 8H). Meanwhile, the results obtained by means of the flow cytometry technique revealed that NEDD4 overexpression lowered the rate of apoptosis of the CFDP1‐downregulated Hep3B cells (Figure 8I). In summary, the above data suggest that CFDP1 promotes the malignancy of HCC cells via the NEDD4/PTEN/PI3K/AKT pathway.

## 4 DISCUSSION

High‐throughput sequencing technology provides an excellent tool for clinical research.^14^ Genes that correlate with a variety of phenotypes could be screened.^15^ However, following each search, a large group of genes is identified. Therefore, the aim of this study is to find out the critical genes. In this article, we obtained samples from patients undergoing HCC surgery and obtained a list of phenotypes, including survival. With the help of the WGCNA method, we discovered a set of genes correlated with overall survival and determined the gene most associated with survival, CFDP1, through LASSO regression. Recent studies have shown that CFDP1 is inextricably correlated with the onset and progression of a wide range of diseases. Research has shown that CFDP1 was identified as a new candidate pancreatic cancer susceptibility gene, and results demonstrated a remarkable reduction in overall survival among the patients with highsss expression.^16^ In a CRISPR‐Cas9‐based study in gastric adenocarcinoma, a series of genes associated with gastric adenocarcinoma prognosis were identified and validated. The findings demonstrated that CFDP1 can independently serve as a prognosticator of survival among patients with gastric adenocarcinoma, which could offer a conceptual framework for targeted therapy of patients after surgery.^17^ Similarly, it has been reported that 16 new loci affecting lung function have been identified based on genome association and large‐scale follow‐up, and CFDP1 is one of them. The results show that the identification of these 16 new sites may help to deeply understand the molecular mechanism of regulating pulmonary function and provide molecular targets for the treatment of pulmonary function decline in the future.^18^ In a multidisciplinary study combining cell biology, reverse genetics, and biochemistry, the in vivo characterization of the function of CFDP1 protein in human cells were reported. The results showed that CFDP1 binds to chromatin and interacts with subunits of SRCAP chromatin remodeling complex. In HeLa cells, RNAi mediated loss of CFDP1 affects chromosome organization, SMC2 agglutinin recruitment, and cell cycle progression. That study provides new insights into the chromatin function and mechanism of CFDP1 and helps us understand the link between epigenetic regulation and the development of complex developmental disorders in human.^19^ At the same time, in a study based on the molecular basis of Williams–Beuren syndrome (WBS), they found that the transcription of CFDP1 gene is directly regulated by the TFII‐I transcription factor family, which provides a basis for further research on the facial dysmorphism of WBS caused by haploidy deficiency of GTF2I and GTF2IRD1.^20^ It has been reported that CFDP1 loci are associated with putative signaling pathways and gene expression in whole blood, monocytes, and myocardial tissue. The results of that study provide insights into the structure, function, and underlying genetic structure of the heart, and provide a basis for further functional study.^21^ Similarly, in the reports about the function development of teeth, CFDP1 function is considered to be the tooth morphogenesis process necessary for cell survival and differentiation, the results showed that affect CFDP1 can adjust the organ culture of tooth size, and in the use of antibodies to inhibit CP27, teeth organ base collapse, apoptosis is significantly increased.^22^

However, to date, the function of CFDP1 in HCC has never been reported. Therefore, it is necessary to clarify the specific role and clear molecular mechanisms of CFDP1 in the onset and progression of HCC. Different methods including WGCNA, western blot TCGA, IHC, qRT‐PCR, and GEPIA were employed for the purpose of measuring the expression levels of CFDP1. The overexpression of CFDP1 in tumor and adjoining normal samples of HCC patients and HCC cell lines was also observed. Clinical data were collected and analyzed. Shorter disease‐free survival (hazard ratio = 1.8; *p* = 0.0099) and poorer overall survival (hazard ratio = 2; *p* = 0.012) were recorded for patients characterized by high levels of CFDP1 expression and experiencing HCC. The results revealed that CFDP1 expression strongly correlated with poor clinicopathological characteristics. Large tumor size increased the extent of microvascular infiltration, and high TNM and Edmonson staging were observed under these conditions. Functional analysis showed that up‐regulation of CFDP1 contributed to the enhanced ability of the HCC cells to proliferate, migrate, invade, and metastasize. Immunofluorescence and western blotting results showed that the upregulation of CFDP1 could promote EMT (for HepG2 cells). Coincidentally, in the HepG2‐CFDP1 overexpression group, promoted EMT may be responsible for a greater proportion of distant lung metastases. We observed that the up‐regulation of CFDP1 inhibited the rate of apoptosis of HCC cells. These findings suggested that CFDP1 could increase the malignant behavioral patterns of HCC cells via the mechanism of modulating the apoptosis in the cells.

We used the Enrichment Analysis, GSEA, and GeneCards tools found that CFDP1 cooperated with NEDD4, which promoted the growth and migration of the HCC cells through the PTEN/PI3K/AKT pathway. It is well known that the neural progenitor expression and developmental downregulation gene 4 (NEDD4) is homologous to the carboxy‐terminal E3 ubiquitin ligase of E6‐AP which plays a role in the ubiquitination of the tumor suppressor gene PTEN, and this results in proteasome degradation and nuclear translocation.^23^ Meanwhile, NEDD4 regulates a large number of membrane proteins through its ubiquitination activity and ability to mediate endocytosis.^24^ Specifically, abnormal expression of NEDD4 may lead to the malignancy of tumor.^25^ Research reports have indicated that NEDD4 is expressed in many tumor types, such as gastric cancer, lung cancer, and colorectal cancer.^25^, ^26^, ^27^, ^28^, ^29^, ^30^ In hepatocellular carcinoma, NEDD4 has been reported to promote hepatocyte growth and migration through the PTEN/PI3K/AKT signaling pathway.^31^ The phosphoinositol 3‐kinase (PI3K) ‐protein kinase B (Akt) signaling pathway is overactivated in most cancers. Deleting the phosphatase and tensin homolog (PTEN) on chromosome 10 has been demonstrated to be a central negative modulator of the PI3K‐AKT signal transduction and is caused by the dephosphorylation of inositol phosphate (PI) and P3 and inhibition of downstream signaling.^32^, ^33^, ^34^, ^35^ Studies have illustrated that miR‐20a induces cellular radio‐resistance by activating the PTEN/PI3K/AKT signaling pathway in HCC, suggesting that miR‐20a/PTEN/PI3K/AKT may serve as a research target in the development of efficacious treatment approaches for HCC^36^. Analysis of the data presented in the present research reveals that CFDP1 cooperates with NEDD4, subsequently promoting the growth and migration of the HCC cells via the PTEN/PI3K/AKT pathway. Thus, a novel mechanism involving CFDP1 and associated with tumor progression was identified. The carcinogenic effect of CFDP1 was significantly rescued when the CFDP1‐downregulated Hep3B‐sh1 cells were transfected with LV‐NEDD4. According to our data, overexpressed NEDD4 may enhance proliferative, migratory, invasive, and metastatic capacities of the CFDP1‐downregulated Hep3B‐sh1 cells. They can also inhibit the apoptosis of tumor cells. To the best of our knowledge, this is the first report on the carcinogenic role and related mechanisms of CFDP1 in HCC. However, further investigations are necessary so as to further investigate the mechanism associated with CFDP1 that results in HCC tumor genesis and development.

In conclusion, it was confirmed that overexpression of CFDP1 was observed in HCC cell lines and tissues. The overexpression of CFDP1 was correlated with unfavorable clinicopathologic characteristics and low relapse‐free survival. It also influenced the overall survival of the HCC patients. A series of functional tests were conducted to reveal the carcinogenic role of CFDP1. Results from in vivo and in vitro studies revealed that mechanistically, CFDP1 enhanced malignancy via the NEDD4‐mediated PTEN/PI3K/AKT pathways. The results can potentially help in providing a therapeutic target for HCC.

## AUTHOR CONTRIBUTIONS

Chunping Jiang, Zhongxia Wang, and Yan Zhou conceived the project and designed the study. Yan Zhou, Jiannan Qiu, Peng Wang, Ding Ma, and Lili Hu carried out experiments in vivo and vitro. Yan Zhou accomplished the manuscript. Siyuan Liu collected clinical samples. Guang Zhang and Yin Cao conducted data analysis. Chunping Jiang, Junhua Wu, and Zhongxia Wang put forward many suggestions. Chunping Jiang and Yan Zhou revised the manuscript. All authors have read and approved the final version to be published.

## CONFLICT OF INTEREST

All authors of this study confirm that they have absolutely no conflict of interest in this manuscript.

## INFORMED CONSENT

This study was approved by the Ethics Committee of the Drum Tower Clinical College of Nanjing Medical University (2016–057‐01) and conducted in accordance with the Helsinki Declaration and government policy. All participants signed a written informed consent form.

## Data Availability

All the data obtained and analyzed during our research can be provided by the corresponding author upon reasonable request.
